# Gall Bladder Wall Thickening in Dengue Fever – Aid in Labelling Dengue Hemorrhagic Fever and a Marker of Severity

**DOI:** 10.7759/cureus.11331

**Published:** 2020-11-04

**Authors:** Benish Adil, Arshad Rabbani, Sualeha Ahmed, Imran Arshad, Muhammad Ali Khalid

**Affiliations:** 1 Internal Medicine, Benazir Bhutto Hospital, Rawalpindi, PAK

**Keywords:** dengue fever, dengue hemorrhagic fever, dhf, gall bladder wall thickness

## Abstract

Introduction

Dengue fever is a mosquito-borne viral disease spread by the bite of the *Aedes aegypti* mosquito. Dengue epidemics have contributed to a great economic burden, especially in South-East Asia. This study aimed to determine gall bladder wall thickness (GBWT) in patients with dengue fever, assess its sensitivity and specificity to identify dengue hemorrhagic fever, and also compare gall bladder wall thickening (GWBT) with platelets, hematocrit, and leucocyte count.

Materials and methods

This prospective observational study was conducted in the dengue ward of Benazir Bhutto Hospital, Rawalpindi, Pakistan, from September 2019 to January 2020, i.e., four months. Patients admitted to the dengue ward diagnosed as seropositive and provided consent were enrolled into the study. Laboratory investigations (blood complete picture, liver function tests, renal function tests) were collected and recorded. Ultrasonography was performed on admission and subsequently during a hospital stay. Patients were divided into two groups: those with gall bladder wall thickness ≤ 3mm and ˃3mm. All data were entered and analyzed on SPSS version 24 (IBM Inc., Armonk, USA).

Results

Out of 180 patients, 122 (67.8%) were male, and 58 (32.2%) female. The mean age was 33 ± 13 years. One hundred and six patients (58.9%) were diagnosed with dengue fever, 68 (37.8%) - dengue hemorrhagic fever, and six (3.3%) - dengue shock syndrome. The most common finding was gall bladder wall thickness ˃3mm (69/180; 38.3%) followed by ascites (38.1%). Sixty-two patients out of 69 (89.9%) with GBWT ˃3mm were managed as dengue hemorrhagic fever (p=0.000). Alanine transaminase (ALT), platelet, and total leukocyte count (TLC) were associated positively with an edematous gall bladder wall (p<0.005). The mean gall bladder wall thickness for dengue hemorrhagic fever was 6.4mm ± 2.5 mm. A GBWT value of 3.5mm was found to have 94.6% specificity and 91.2% sensitivity.

Conclusion

Gall bladder wall edema is strongly correlated with dengue hemorrhagic fever. Hence it should be assessed in all patients with dengue fever.

## Introduction

Dengue fever is a viral hemorrhagic fever transmitted by *Aedes aegypti* and *Aedes albopictus*. It poses a great economic burden worldwide and specifically in South East Asia. WHO estimated that 50-100 million dengue infections occur worldwide annually, with a 30-fold increase in global incidence observed over the past five years [1].

Almost 75% of the global population exposed to dengue live in the Asia Pacific, and overall expansion in dengue cases have been noted in the past decade [2, 3]. Since 2005, Pakistan has been having major and minor outbreaks of dengue fever/ dengue hemorrhagic fever annually, caused mainly by dengue virus (DENV)-2 and DENV-3, although DENV-1 and DENV-4 have also been isolated in recent years [4-6]. A major dengue epidemic was recorded in the city of Lahore in 2011, with 21,685 confirmed cases and 350 deaths [7].

In recent years, dengue fever epidemics have shown varied clinical presentations, thus delaying diagnosis and treatment [7]. Typical dengue fever is characterized by high-grade fever, musculoskeletal pain, retro-orbital pain, headache, joint pain, nausea, vomiting, and morbilliform rash. Fever, headache, and abdominal pain are common manifestations [8]. 

In a small proportion of cases, the virus causes increased vascular permeability that leads to a bleeding diathesis or disseminated intravascular coagulation (DIC) known as dengue hemorrhagic fever (DHF) [9]. Secondary infection by a different dengue serotype has been confirmed as an important risk factor for the development of DHF [10, 11]. 

It is important to identify DHF at its initial stages so that proper management can be instituted timely and morbidity and mortality prevented [12]. Clinically many parameters can be used to predict the onset of capillary leakage in DHF, including thrombocytopenia, hemoconcentration, and presence of free fluid in the peritoneal/pleural cavity detected on ultrasound.

A few studies have reported that ultrasound plays an important role as a prognostic indicator in assessing patients at risk for progressing to the critical phase by measuring gall bladder wall thickness [13]. In contrast to existing markers such as hematocrit, serial ultrasonography may better identify patients at risk for the development of severe dengue, but there is a paucity of literature to support this notion.

The rationale of this study is to assess the credibility of the non-invasive investigation, i.e., ultrasonography in detecting early dengue hemorrhagic fever, in comparison to other clinical parameters used to recognize the condition and allowing the treating physician to identify the condition at the earliest and manage accordingly.

## Materials and methods

This prospective observational study was carried out to determine the association of gall bladder wall thickness in patients with dengue fever that will help identify patients with severe illness and predict the onset of dengue hemorrhagic fever. A total of 1,200 patients with dengue fever presented to Benazir Bhutto Hospital, Rawalpindi, Pakistan, during the peak of the dengue epidemic in 2019 observed in Rawalpindi from September 2019 to January 2020. Approval for the study was obtained from the ethical committee of the hospital. Data were collected during this epidemic prospectively, and analysis was done after the epidemic was over.

Selection of study participants

From this epidemic, patient enrollment was done via stratified sampling. The sample size was calculated to be 180. Both males and females above 16 years of age who had a fever of more than two days and were confirmed to have dengue fever on the presence of non-structural protein 1 (NS1) or immunoglobulin M (IgM) and immunoglobulin G (IgG) antibodies against dengue were included in the study. Participants with only dengue IgG antibodies positive (whose presence alone signifies the previous infection) were excluded from the study. Similarly, pregnant females, those with intrinsic gall bladder disease, and those with a history of congestive cardiac failure or chronic liver disease were also excluded from the study. Consent was taken from all included study participants after explaining to them the nature and purpose of the research. Clinical history was taken regarding symptoms and recorded. All data of the patients were recorded on performas.

Laboratory analysis

Demographic details and variables of the patients who fulfilled the inclusion criteria were systematically recorded after informed consent was obtained. These included age, sex, illness duration, initial diagnosis on admission, and final diagnosis upon discharge. Blood complete picture (CP) was performed on admission and also during in-hospital management. Other laboratory parameters conferred to the severity of illness like alanine transaminases (ALT) and creatinine were recorded during treatment.

Dengue serology and classification 

Patients who had NS1 or IgM or both positive were considered confirmed diagnoses of dengue fever with primary infection. Those with additional IgG positive were considered as having a secondary infection. Patients were classified according to WHO 1997 Dengue Classification into dengue fever (DF), dengue hemorrhagic fever (DHF) (conferring to plasma leakage), and dengue shock syndrome (DSS) (conferring to hemodynamic compromise and multi-organ failure). Criteria for DHF, as given by WHO 1997 guidelines, included thrombocytopenia of <100,000/mm^3^, hemorrhagic manifestations, and evidence of plasma leakage as shown by hemoconcentration, i.e.,>20% rise in hematocrit from the baseline, or presence of ascites, pleural effusion. DSS criteria were defined as DHF along with circulatory failure manifested as narrow pulse pressure (<20mmHg), cold peripheries, and rapid, weak pulse [14]. This is shown in Table 1.

**Table 1:** WHO 1997 dengue classification Source: [14]

Dengue classification criteria	
Dengue fever (DF)	
Acute febrile illness with two or more of the following	Headache
	Retro-orbital pain
	Myalgia
	Leukopenia
	Arthralgia
	Rash
	Supportive serology or occurrence at the same location and time as other confirmed cases of dengue fever
Dengue hemorrhagic fever (DHF)	
All of the following must be present	Fever or history of acute fever, lasting 2-7 days, occasionally biphasic
	Hemorrhagic manifestations: (positive tourniquet test, petechia, ecchymosis, purpura or bleeding from the mucosa, hematemesis/melena
	Thrombocytopenia (<100000 platelets/mm^3^)
	Evidence of plasma leakage due to increased vascular permeability
Dengue shock syndrome (DSS)	
DHF with hypotension for age or narrow pulse pressure (<20mmHg), plus	Rapid and weak pulse
	Cold clammy skin, restlessness

Ultrasonography

Abdominal ultrasound was done daily for all study participants on admission and during treatment. Ultrasound was done on an empty stomach (fasting for six hours or more). This investigation aimed to detect plasma leakage in the form of ascites, pleural effusion, and edematous gall bladder wall and also to detect any visceromegaly. Hepatomegaly was defined as the right lobe of the liver measuring >15cm in the midclavicular line, and splenomegaly was defined as the spleen measuring >12 cm. Gall bladder wall thickness was estimated by placing calipers over the anterior abdominal wall. All ultrasound examinations were performed by two skilled and experienced radiologists who were blinded about the clinical and laboratory parameters of the study participant. The average values of their findings were used for analysis.

Data analysis

All data collected were entered on SPSS version 24 (IMB Inc., Armonk, USA). Variables like age, gender, diagnosis, serology, ultrasound findings were represented as percentages. Mean values of platelets, alanine transaminase, leucocyte count, and creatinine were calculated. Study participants were grouped into two groups: GBWT ≤3mm and GBWT more than 3mm. The comparison of GBWT with diagnosis, platelets, leucocyte count, and hematocrit was assessed by the Chi-square test; a p-value of less than 0.005 was considered statistically significant. To identify the sensitivity and specificity of GBWT for the diagnosis of DHF, the receiver operating characteristic (ROC) curve was plotted. 

## Results

Our study included 180 patients, of which 122 (67.8%) were male, and 58 (32.2%) were female. The mean age was 33 years ± 13 years, ranged from 16-73 years. The most common clinical manifestation on presentation was fever (100%), followed by abdominal pain (50%). Significant bleed was observed in only six study participants; four out of six had epistaxis (66.7%) requiring nasal packing, one had significant gum bleed and one had hematemesis. One hundred and six patients out of 180 (58.9%) were managed as DF, 68 (37.8%) had DHF, and six (3.3%) had DSS. The physical characteristics of the study participants are shown in Table 2.

**Table 2:** Demographics of the study participants

Parameter		Frequency
Age (years)	11-20	36 (20%)
	21-30	56 (31.1%
	31-40	47 (26.1%)
	41-50	20 (11.1%)
	51-60	13 (7.2%)
	61-70	6 (3.3%)
	70 above	2 (1.1%)
Symptoms	Headache	85 (47.2%)
	Fever	180 (100%)
	Abdominal pain	90 (50%)
	Nausea/vomiting	83 (46.1%)
	Retro-orbital pain	59 (32.8%)
	Bleeding	6 (3.3%)
Diagnosis	Dengue fever	106 (58.9%)
	Dengue hemorrhagic fever	68 (37.8%)
	Dengue shock syndrome	6 (3.3%)

The serology testing revealed that 65 (36.1%) patients were NS1 positive only, five (2.8%) were IgM positive only, 32 (17.8%) were NS1 and IgM positive, 25 (13.9%) were NS1 and IgG positive, 16 (8.9%) were IgM and IgG positive and 37 (20.6%) were triple positive. So, our study comprised of 78 (43.3%) secondary dengue infection cases. The mean values of other lab parameters are presented in Table 3.

**Table 3:** Serology and laboratory parameters NS1 - non-specific antigen 1; IgM - immunoglobulin M; IgG - immunoglobulin G; TLC - total leucocyte count; Plt - platelets; HCT - hematocrit; ALT- alanine aminotransferase

Parameters		Frequency
Primary dengue infection	NS1 only	65 (36.1%)
	IgM only	5 (2.8%)
	NS1 and IgM	32 (17.8%)
Secondary dengue infection	NS1 and IgG	25 (13.9%)
	IgM and IgG	16 (8.9%)
	NS1, IgM and IgG	37 (20.6%)
Laboratory parameters (mean values)	TLC (x10^3^ cells/µl)	4.3 ± 1.8
	Plt (x10^3^ cells/µl)	69.3 ± 31.9
	HCT (%)	41.0 ± 4.3
	ALT (IU/l)	80.6 ± 193.9 (range: 2176.0)
	Creatinine (mg/dl)	0.9 ± 0.25

Ultrasonography revealed the most common finding to be gall bladder wall thickness (>3mm) which was seen in 69 (38.3%) study participants. Next most common finding was ascites seen in 38 (21.1%) patients. Other findings were splenomegaly in four (2.2%), pleural effusion in three (1.7%) and both ascites and pleural effusion in two (1.1%) study participants. Mean GBWT was 6.4mm ± 2.5 mm in patients managed as DHF and 8.3mm ± 2.1mm in patients managed as DSS. Forty (63.5%) study participants, who had a thickened gall bladder wall on admission, did not show any other radiological signs of plasma leakage on admission, i.e., pleural effusion or ascites (p≤0.001). Ultrasound findings on admission and during hospital stay are given in Table 4. GBWT and its association with the severity of illness and laboratory parameters are given in Tables 5-6. As evident from Table 5, 62 out of 69 patients with GBWT >3mm (89.9%) were managed as DHF while only one of these patients (1.4%) was managed as DF (p<0.001). All patients with DSS were actually found to have GBWT >3mm.

**Table 4:** Ultrasound findings of the patients GBWT - gall bladder wall thickness

Ultrasound finding	On admission	Subsequent
Pleural effusion	1 (0.6%)	3 (1.7%)
Ascites	22 (12.2%)	38 (21.1%)
Pleural effusion + ascites	3 (1.7%)	2 (1.1%)
Splenomegaly	4 (2.2%)	4 (2.2%)
GBWT >3mm	63 (35%)	69 (38.3%)

**Table 5:** Comparison of GBWT with gender and diagnosis GBWT - gall bladder wall thickness; DF - dengue fever; DHF - dengue hemorrhagic fever; DSS - dengue shock syndrome

	GBWT ≤3mm (n=111; 61.7%)	GBWT>3mm (n=69; 38.3%)	Total (n= 180; 100%)	p-value
	Gender			
Male	83 (74.8%)	39 (56.5%)	122 (67.8%)	0.014
Female	28 (25.2%)	30 (43.5%)	58 (32.2%)	
	Diagnosis based on WHO 1997 dengue classification			
DF	105 (94.6%)	1 (1.4%)	106 (58.9%)	<0.001
DHF	6 (5.4%)	62 (89.9%)	68 (37.8%)	
DSS	0 (0%)	6 (8.7%)	6 (3.3%)	

**Table 6:** Comparison of GBWT with age and laboratory parameters GBWT - gall bladder wall thickness; ALT - alanine aminotransferase; TLC - total leucocyte count; HCT - hematocrit

	GBWT ≤3mm (n=111; 61.7%)	GBWT >3mm (n= 69; 38.3%)	Total (n=180; 100%)	p-value
Age (years)	32.7 ± 13.2	34.3 ± 13.5	33 ± 13	0.431
Mean ALT (IU/l)	32.7 ± 1.7	157 ± 35.9	80.6 ± 193.9	<0.001
Mean platelet count (x10^3^ cells/µl)	82.6 ± 31.9	48.1 ± 17.3	69.3 ± 31.9	<0.001
Mean TLC (x10^3^ cells/µl)	4.0 ± 1.5	4.9 ± 2.1	4.3 ± 1.8	0.002
Mean HCT (%)	40.7 ± 4.3	41.5 ± 4.4	41.0 ± 4.3	0.258
Mean creatinine (mg/dl)	0.8 ± 0.2	1.0 ± 0.2	0.9 ± 0.25	<0.001

**Figure 1:**
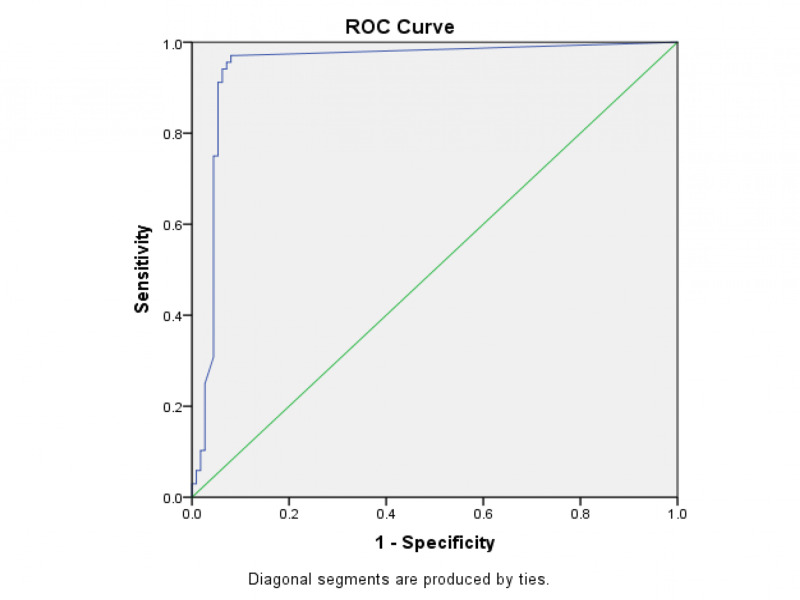
ROC curve for specificity and sensitivity of gall bladder wall thickening for diagnosing dengue hemorrhagic fever Area under curve = 0.944 ROC - receiver operating characteristic

A ROC curve was plotted to identify a cut off value for GBWT in DHF, as shown in Figure 1. A GBWT value with 94.6% specificity and 91.2% sensitivity was found to be 3.5mm. The area under the curve is 0.944, which is significant.

## Discussion

Dengue hemorrhagic fever and dengue shock syndrome have been notorious for resulting in mass deaths on a large scale, yet being preventable at the same time. The antibodies generated by the four serotypes of dengue virus (DENV-1, DENV-2, DENV-3, DENV-4) share little cross-immunity; hence secondary infections are associated with a more severe disease manifestation. Identifying the relation of one important yet under-rated indicator of DHF/DSS, i.e., gall bladder wall thickness, with other parameters of severity for this disease was the main focus of our study.

The results of our study favored the fact that GBWT is directly related to disease severity. The mean age of our study is 33 ± 13 years, which shows that our study focused on the younger population. Males comprise most of the study participants (68%), probably due to their increased outdoor activities. Most of our patients had simple/classical dengue fever (58.9%), and most of them had a primary infection (56.7%), showing a positive correlation between these two. Dayanand KR, et al. reported that the most common ultrasound finding in seropositive is gall bladder wall thickness (86.5%) followed by ascites (41.7%) [15]. Similar findings were observed in our study also. Our study shows that 62 out of 69 patients (89.9%, p<0.001) with DHF had a thickened gall bladder wall greater than 3mm. Similar significance of GBWT in plasma leakage is depicted in various other studies. Nainggolan L et al. also documented that 30 out of 46 patients (65.2%) with plasma leakage had GBWT [16]. The lesser percentage might be because the cited study performed ultrasonography to detect GBWT soon on admission, i.e., the third day, while our study analyzed the mean value of GBWT obtained on admission and then performed subsequently during the hospital stay. Khurram M, et al. states that only 23.7% of patients with DHF had GBWT [17].

Mean GBWT for patients with plasma leakage, i.e., DHF in our study, was calculated to be 6.4mm ± 2.5 mm in patients managed as DHF and 8.3mm ± 2.1mm in patients managed as DSS. These values are comparable to those quoted by Nainggolan L, et al., i.e., 3.30mm [16]. In a study conducted by Parmar JP, et al., GB wall thickness was 3.32 mm in DF without warning signs, 4.95 mm in DF with warning signs, and 8.80 mm in severe DF, which is in line with our study results [18].

Our study can also establish the correlation between GBWT with other severity markers, i.e., alanine transaminases, platelets, total leucocyte count, and hematocrit. In our study, a thickened GBWT is strongly associated with a raised ALT: patients with thickened gall bladder wall had a mean ALT of 157 ± 35.9 IU/l. Fernando S et al. report that 69% of their study participants with non-severe dengue had an ALT between 40-160IU/l [18]. A statistically significant association between elevated liver enzymes and the fall in platelets and other features of DHF are also reported [20], but there is a paucity of direct correlation of GBWT with ALT in DHF. In our study, mean value of platelets in patients with GBWT is 48.1 ± 17.3 x103 cells/µl (p-value <0.001). Manam G et al., Bajaj S et al., and Nagolu H et al. support this fact statistically; however, Tavares MdA et al. show no association of GBWT with platelet count or other hematological parameters [21-24]. According to our study results, hematocrit shows no statistically significant association with GBWT (p=0.258), which is in line with the recent evidence that hematocrit is a poor indicator of the severity of dengue infection [25]. As per our study results, in 63.5% of patients, GBWT preceded the development of ascites or pleural effusion; the same has been reported by Vedaraju KS, et al., that development of GBWT may herald the onset of plasma leakage in patients with dengue infection [26].

Our study lacks post-discharge follow-up ultrasonography to look for timings of normalization of gall bladder wall edema. Also, GBWT patterns and their association with disease severity were not covered in our study, providing future research opportunities in this domain. To provide a stronger association between GBWT and DSS, a greater number of patients with DSS should have been included in the study. 

## Conclusions

Dengue fever is a preventable disease with basic and supportive management only. Yet the large scale mortality that it causes in the form of epidemics, warrant a thorough knowledge about its prevention, risk factors and severity predictors. Our study results are able to provide a strong correlation between GBWT and disease severity. The authors hence recommend that among other clinical and laboratory parameters, gall bladder wall thickness should also be assessed in every patient with dengue infection, and should never be overlooked or underestimated.
